# Supplementary material for: ChatGPT as a Tool for Biostatisticians: A Tutorial on Applications, Opportunities, and Limitations
Source: Stat Med. 2025 Oct 23;44(23-24):e70263. doi: 10.1002/sim.70263 (PMC12548020; doi:10.1002/sim.70263)
Supplement: Supplementary file 1 — Data S1. Supporting Information. [file SIM-44-0-s001.zip › Supplementary Materials Additional.zip!/3.1_Systematic_Reviews_and_Meta_aspects/3.1_Data Extraction - o4-mini - Chat 6.pdf]

Use Case Meta-Analysis - Data Extraction - o4-mini - Chat 6

Rudloff et al. - 2009 - Concurrent lobular neoplasia increases the risk of ipsilateral breast cancer recurrence in situ treated with breast-conserving therapy.pdf
PDF

Vidali et al. - 2012 - Conservative treatment of breast ductal carcinoma in situ results of an Italian multi-institutional.pdf
PDF

The aim is to perform a meta-analysis on total ipsilateral local recurrence rates at 10 years in cases of ductal carcinoma in situ.

Data inclusion rules are: To ensure the validity of the follow-up data, the results should be as close to 10 years follow-up (minimum) as the information in each study allows. If there is more than one publication from an institution or group, the most recent study with appropriate follow-up will be used to extract 10-year data.

Please provide from each of the two attached studies:

- the rate of ipsilateral local recurrence for conservative surgery with additional radiotherapy (no other interventions or drugs allowed) after 10 years

- the number of eligible patients

- period of data collection

- duration of follow-up

- type of study

- age of patients (mean or median, maximum and minimum)

- treatment modalities

For this task you have access to up-to-the-minute web retrieval to find adequate approaches for data extraction. When answering the task above please proceed as follows:

1. Fetch the most relevant sources.

2. Show your step-by-step reasoning.

3. At the end, give me a concise answer citing the sources you used.

4. Please provide a dataframe with one entry for each study, and column names "study", "rate", "n_patients", "period", "follow_up", "study_type", "age", and "treatment".

Step-by-step reasoning

1. I retrieved the Rudloff et al. (2009) study and located the actuarial 10-year ipsilateral recurrence rates stratified by receipt of radiotherapy, the study period, follow-up duration, patient age, and treatment details .
2. I retrieved the Vidali et al. (2012) multi-institutional Italian study and identified the 10-year local recurrence rate, total number of patients, accrual period, length of follow-up, study design, patient age, and treatment regimen .
3. I extracted and organized these data into the requested columns.

Concise summary of key data

study	rate	n_patients	period	follow_up	study_type	age	treatm[illegible]
Rudloff et al. (2009)	11.9%	294	1991–1995	Median 11 years (range 0–16 yrs)	Retrospective cohort analysis	Median 55 yrs (26–89)	Breast-co[illegible] surgery + external-b[illegible] tangential[illegible]
Vidali et al. (2012)	9.6%	586	Feb 1985–Mar 2000	Median 136 mo (16–292 mo)	Retrospective multi-institutional study	Median 55 yrs (29–84)	Conservat[illegible] surgery + whole-bre[illegible] (median 5[illegible] boost 50%[illegible]

All treatment arms included only surgery and radiotherapy, with no other systemic therapies.

Quellen
